# Supplementary material for: Cis- and trans-regulations of pre-mRNA splicing by RNA editing enzymes influence cancer development
Source: Nat Commun. 2020 Feb 7;11:799. doi: 10.1038/s41467-020-14621-5 (PMC7005744; doi:10.1038/s41467-020-14621-5)
Supplement: Supplementary file 1 — Supplementary Information [file 41467_2020_14621_MOESM1_ESM.pdf]

Supplementary Information

Cis- and *trans*-regulations of pre-mRNA splicing by RNA editing enzymes influence cancer development

Tang and Shen et al.

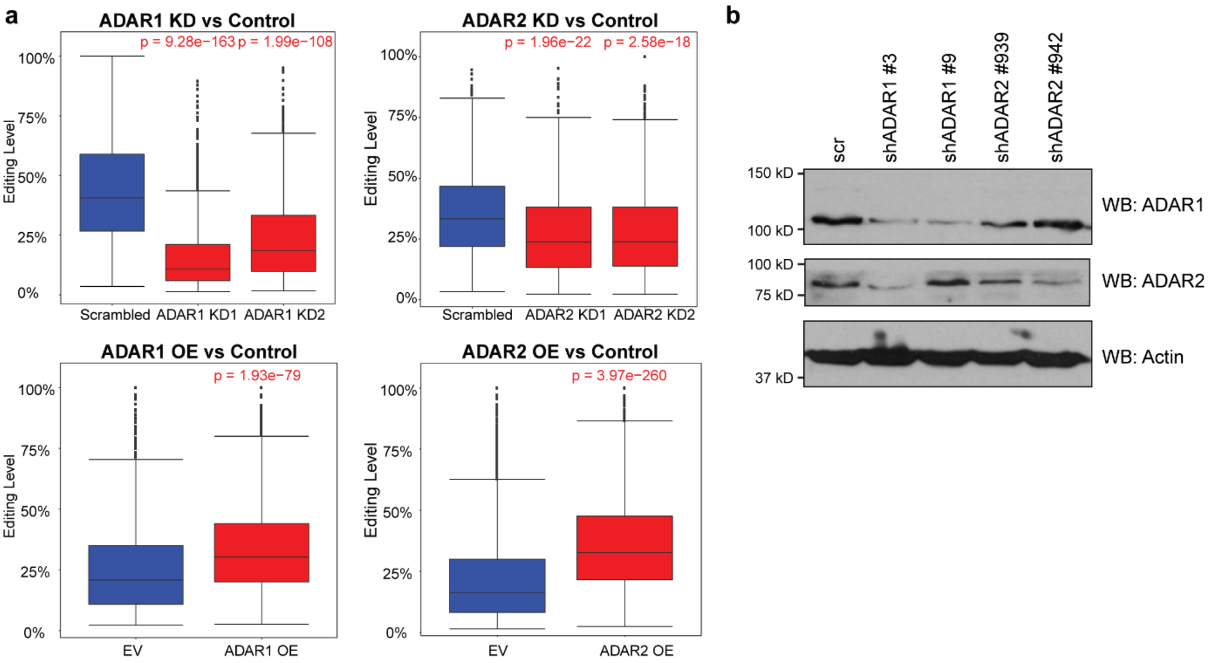

Supplementary Fig. 1. ADARs regulate editing and alternative splicing.

a Box plots illustrating the differential overall editing level in the indicated groups of cells. Data are presented as box plots with median (horizontal line), 25–75 percentile (box) and 5–95 percentile (whisker) for each group and black dots indicate the outliers (Wilcoxon Signed-Rank Test).

b WB analyses of ADAR1 and ADAR2 proteins in HEK293T cells that were transfected with the indicated shRNAs. β-actin (Actin) was used as a loading control.

Source data are provided as a Source Data file.

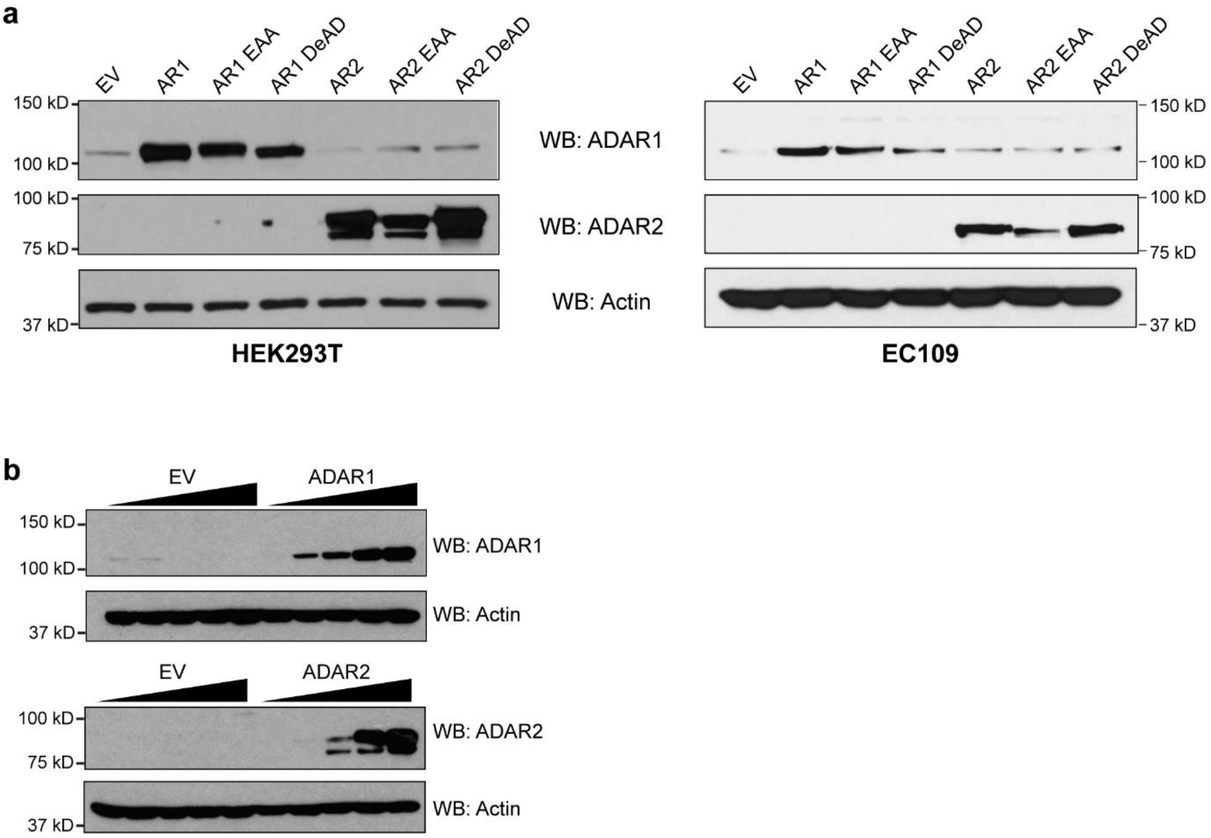

Supplementary Fig. 2. ADAR proteins repress exon inclusion through RNA editing dependent and independent mechanisms.

a WB analysis of ADAR1 and ADAR2 proteins in HEK293T (left) and EC109 (right) cells that were transfected with the indicated expression construct.

b WB analysis of ADAR1 and ADAR2 proteins in HEK293T cells that were transfected with increasing amount of EV, *ADAR1* or *ADAR2* construct. β-actin (Actin) was used as a loading control.

Source data are provided as a Source Data file.

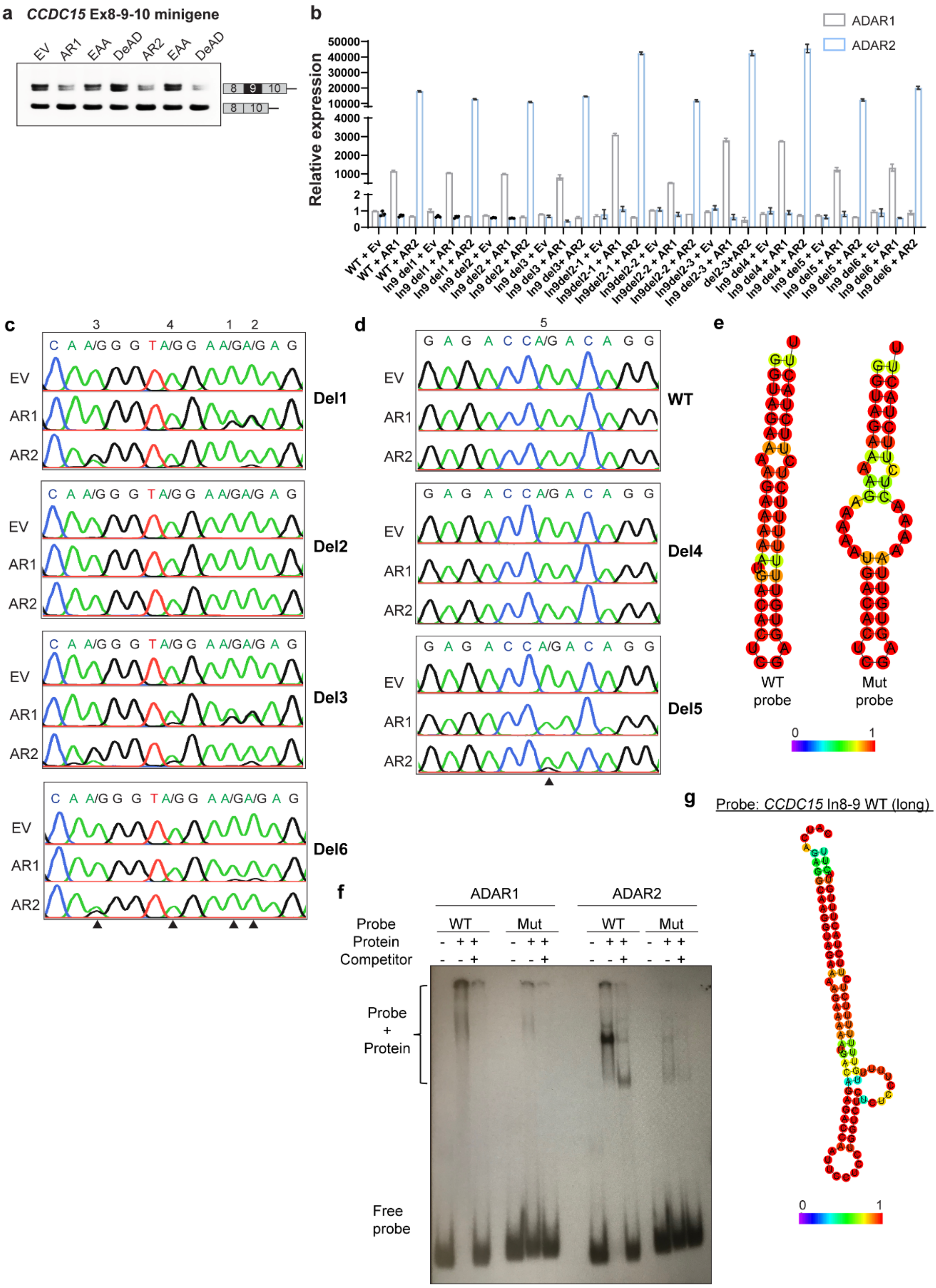

Supplementary Fig. 3. An intronic dsRNA formed between intron 8 and 9 of *CCDC15* is essential for ADARs binding and splicing regulation.

a RT-PCR analysis of exon 9 inclusion of exogenous *CCDC15* transcripts in HEK293T cells that were co-transfected with *CCDC15* minigene and the indicated expression construct.

b QPCR analyses of *ADAR1* and *ADAR2* transcript levels in the samples as described in Fig. 4b. Data are presented as the mean ± S.D. of relative expression derived from qPCR technical replicates from a representative experiment.
c,d Sequence chromatograms illustrate the editing level of the indicated sites (1-5) in HEK293T cells that were co-transfected with the indicated minigene and EV, *ADAR1* or *ADAR2* construct. Black arrowhead indicates editing position.
e Predicted RNA secondary structures of wild-type (WT) and mutated (Mut) *CCDC15* probes used in REMSA analysis (Fig. 4e) by RNAfold. Minimum free energy (MFE) structures drawing encoding base-pair probabilities are shown. Base-pair probabilities are shown by a colour spectrum. Asterisk represents editing site.
f REMSA analysis of the binding of ADAR1 or ADAR2 protein to *CCDC15* transcripts *in vitro*, using ^{32}P-labelled WT or Mut probe as shown in (**e**) and purified ADAR1 or ADAR2 protein. Unlabelled RNA probes were used as competitor.
g Predicted RNA secondary structure of the wild-type *CCDC15* dsRNA probe used in REMSA analysis (Fig. 4g) by RNAfold.
Source data are provided as a Source Data file.

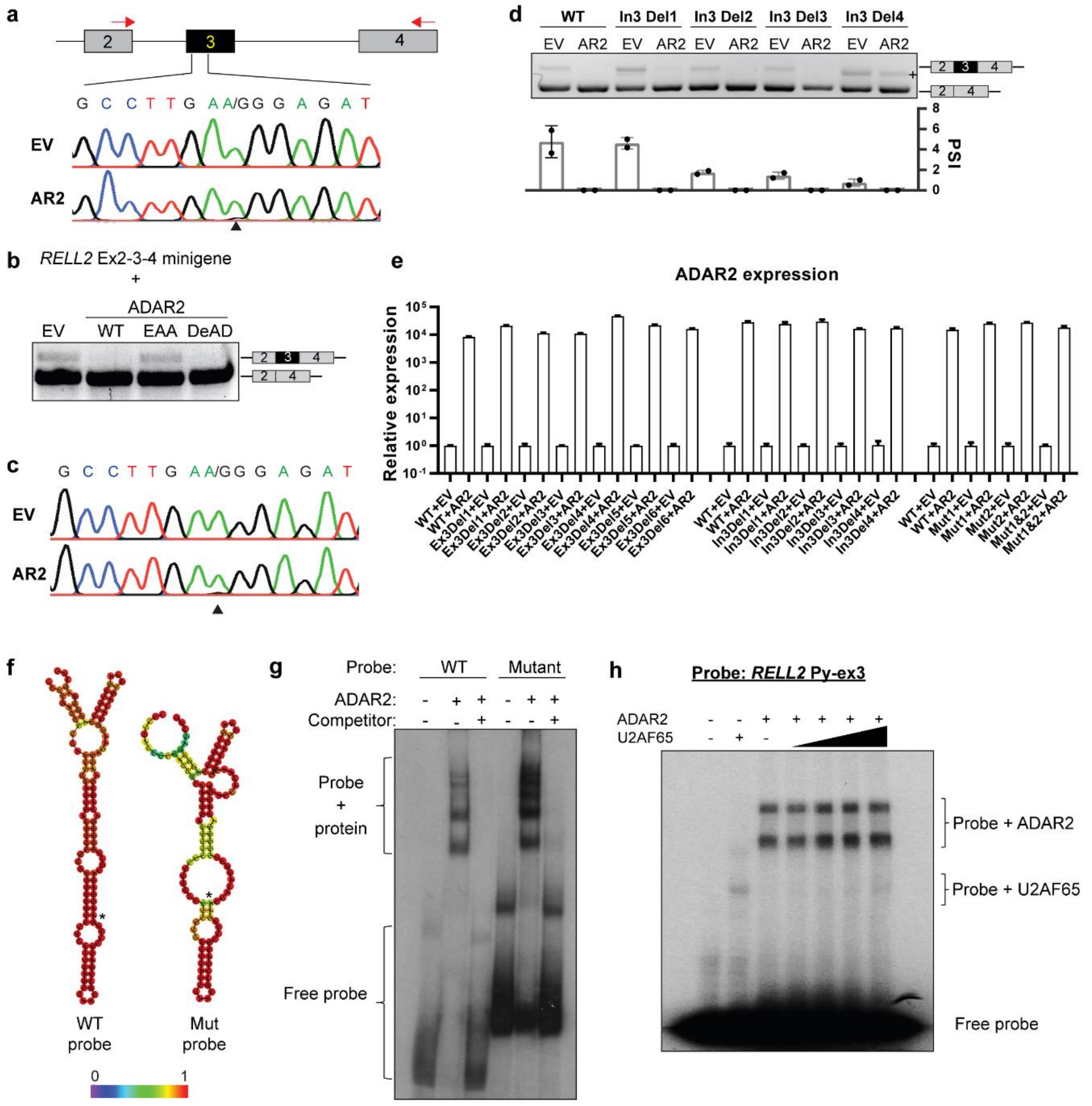

Supplementary Fig. 4. ADAR2 binds to *RELL2* dsRNA formed between exon 3 and the Py-tract.

a Sequence chromatograms illustrate the editing level of an editing site within exon 3 of endogenous *RELL2* transcripts in HEK293T cells transfected with EV or *ADAR2* construct. Black arrowhead indicates editing position. Red arrows show the location of primers used for PCR amplification.

b RT-PCR analysis of exon 3 inclusion of exogenous wild-type *RELL2* transcripts in HEK293T cells that were co-transfected with *RELL2* minigene and the indicated expression construct.

c Sequence chromatograms illustrate the editing level of an editing site within exon 3 of exogenous *RELL2* minigene-derived transcripts in HEK293T cells transfected with EV or *ADAR2* construct. Black arrowhead indicates editing position.

d RT-PCR analysis of exon 3 inclusion of exogenous *RELL2* transcripts in HEK293T cells that were co-transfected with the indicated minigene and EV or *ADAR2* construct (n=2 biological replicates for each). Plus sign indicates a cryptic splice product generated by In3 Del4 mutant. Data are presented as the mean ± S.D. of PSI values from biological replicates. Each dot represents a biological replicate.

e QPCR analyses of *ADAR2* transcript levels in the samples as described in Fig. 5b,d and Supplementary Fig.4d. Data are presented as the mean ± S.D. of relative expression derived from qPCR technical replicates from a representative experiment.

f Predicted RNA secondary structures of wild-type (WT) and mutated (Mut) *RELL2* Py-ex3 probes by RNAfold. Base-pair probabilities are shown by a colour spectrum. Asterisk represents editing site.

g REMSA analysis of the binding of ADAR2 protein to *RELL2* transcripts *in vitro*, using ^{32}P-labelled WT or Mut *RELL2* Py-ex3 probe as shown in (**e**) and purified ADAR2 protein. Unlabelled RNA probe was used as competitor.

h *In vitro* RNA-protein binding assay by UV crosslinking was carried out to examine the binding of ADAR2 to the *RELL2* Py-ex3 dsRNA probe, in response to increasing amount of U2AF65 protein.

Source data are provided as a Source Data file.

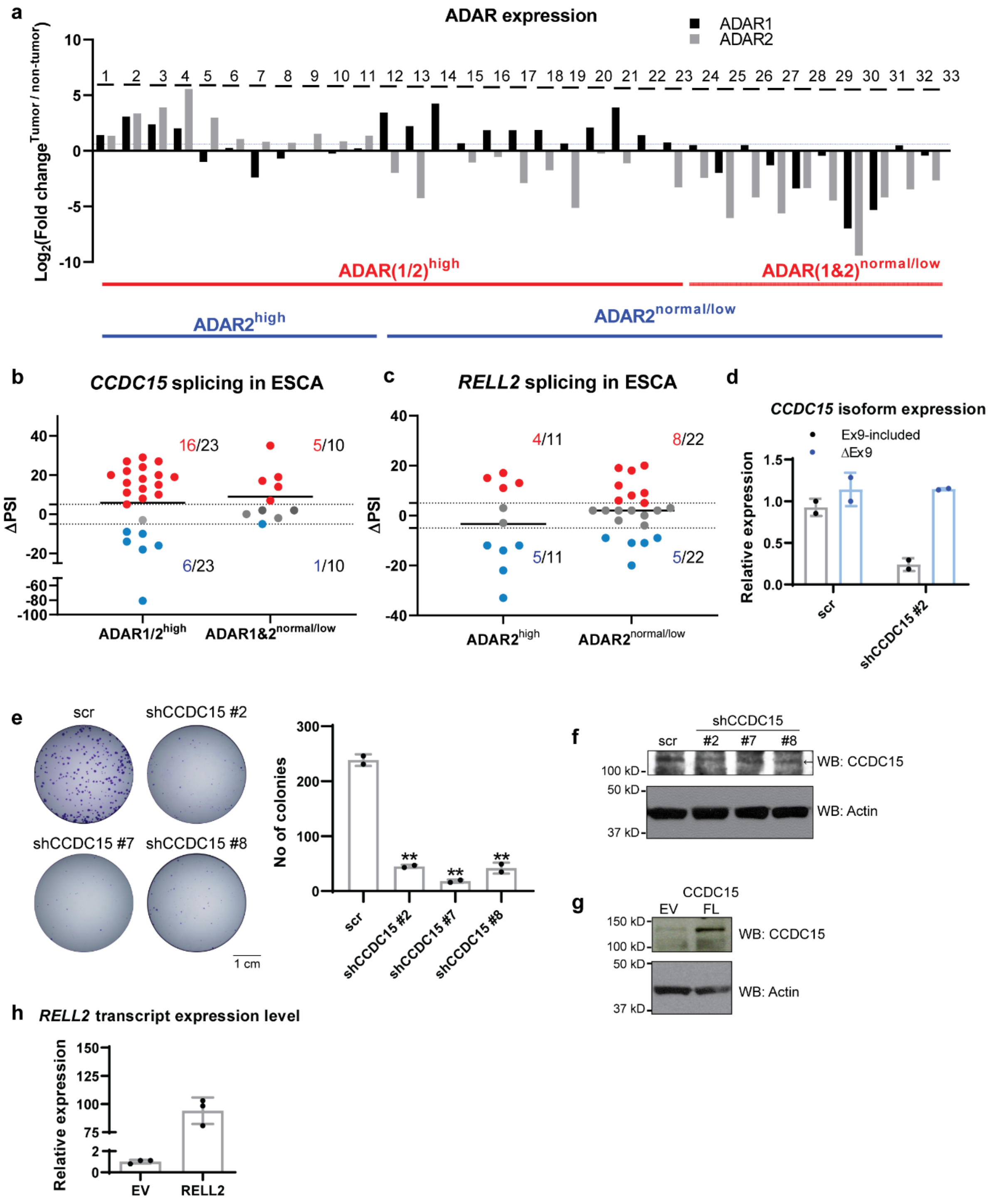

Supplementary Fig. 5. Exon 9-included *CCDC15* isoform is oncogenic.

a QPCR analyses of ADAR1 and ADAR2 expression in 33 matched pairs of primary ESCC and non-tumor (NT) samples. Patients demonstrating ≥1.5-fold higher expression of ADAR1 or/and ADAR2 in tumors than their matched NT tissues were classified as ADAR(1/2)^{high} group; while the remaining cases were included into ADAR(1&2)$^{normal/low}$ group. Patients demonstrating ≥1.5-fold higher expression of ADAR2 in tumors than their matched NT

tissues were classified as ADAR2^{high} group; while the remaining cases were included into ADAR2$^{normal/low}$ group. Dotted line depicts the 1.5-fold cut-off.

b,c Dot plots showing ΔPSI of *CCDC15* exon 9 (**b**) and *RELL2* exon 3 (**c**) in 33 matched pairs of primary ESCC and NT samples, grouped by ADAR1/2 expression level.

d QPCR analysis of expression level of *CCDC15* exon 9-skipped (ΔEx9) or included (Ex9-included) isoform in the indicated cells. Data are presented as the mean ± S.D. of relative expression derived from qPCR technical duplicates from a representative experiment. Each dot represents a technical replicate.

e Quantification of foci formation induced by the indicated cells. EC109 cells were stably transduced with scr, sh*CCDC15* #2, sh*CCDC15* #7 or sh*CCDC15* #8 lentivirus. Data are presented as the mean ± S.D. of duplicate wells from a representative experiment. Each dot represents a biological replicate (n=2 biological replicates for each). Scale bar, 1 cm. Statistical significance is determined by unpaired, two-tailed Student's *t*-test (**, P <0.01).

f,g WB analysis of CCDC15 protein in EC109 cells transduced with different shRNAs (**f**) or *CCDC15* full-length (FL, Ex9-included) expression construct (**g**).

h QPCR analyses of *RELL2* transcript levels in the indicated cells. Data are presented as the mean ± S.D. of relative expression derived from qPCR technical triplicates from a representative experiment. Each dot represents a technical replicate.

Source data are provided as a Source Data file.

Supplementary Table 1: Mapping summary of RNA-Seq reads obtained from EC109 cells with depletion or overexpression of ADAR1 or ADAR2. Over 90% of reads map uniquely to the human genome reference hg19 in all samples.

Sample	Total Reads	Uniquely mapped Reads	Uniquely mapped reads percentage
EC109 scr	239061333	225792765	94.45%
EC109 shADAR1 #3	139290010	132055981	94.81%
EC109 shADAR1 #9	117926188	111406712	94.47%
EC109 shADAR2 #939	150277869	142232700	94.65%
EC109 shADAR2 #942	127013368	120313736	94.73%
EC109 pLenti6	119973548	112592789	93.85%
EC109 pLenti-ADAR1	126014537	118796655	94.27%
EC109 pLenti-ADAR2	150578063	141152621	93.74%

Supplementary Table 2: List of primers and RNA probes

Primers for validation	
CCDC15.Ex8.F	CCTGCATGTTCTCTCCAACG
CCDC15.Ex10.R	CTGTTCTCTAGGGGAGTCTTGG
RELL2.Ex2.F	GAGGACGATGACATGAATGAGG
RELL2.Ex4.R	CTGCCCACAGAGAACACAGTG
ZNF778.Ex2.F	ATCCGTGGGTCAGGAGGAATG
ZNF778.Ex4.R	TACTGAGGCCAGGTTCTCG
ZNF517.Ex2.F	TCACTGTCTGTAGCATCTGCTC
ZNF517.Ex4.R	GCTCCTCTCCTTGCTCCAAT
KLRG1.Ex2.F	GGCCTTCTTGTTCTTGCCTTGTG
KLRG1.Ex4.R	CATCTTCCCACCTCCAGCCAG
AKAP9.Ex18.F	TTCAGTGTCAAGCATGGATGC
AKAP9.Ex20.R	CTCAGGAGGCACATCTTCAGG
Primers for RT-PCR	
CCDC15.Ex8/9.F	GATTTTCTACCAAAATATCAGAAAGTACAC
CCDC15.Ex8/10.F	GATTTTCTACCAAAATATCAGCAACCTG
CCDC15.In8.F	GAGAGAGGCACTTCCTCTGG
CCDC15.In9.R	GACTCCATCAGAAAAAAGCCAC
CCDC15.Q.F	TTTACGAGCCCAAATCCAGG
CCDC15.Q.R	ATGAATGTAGTGCCCGAGTATATG
RELL2.Ex2.F	GAGGACGATGACATGAATGAGG
RELL2.Ex4.R	CTGCCCACAGAGAACACAGTG
RELL2.Q.F	AAGAGGCCTCCACTTGTCC
RELL2.Q.R	GTGTCACCCGGAACCTGC
pcDNAF	GAGACCCAAGCTGGCTAGCGTT
pcDNAR	GAGGCTGATCAGCGGGTTTAAAC
SRSF1.Ex3.F	CACTGGTGTCGTGGAGTTTGTACGG
SRSF1.Ex4.R	GGGCAGGAATCCACTCCTATG
ZDHHC16.Ex7.F	GTGTGGGCCACTATAACCATCG
ZDHHC16.Ex9.R	CTGCACAGGAACCAGAGGTAG

Primers for RIP-qPCR	
CCDC15.In8.3140R	GCTGAGAACAACCTTTGAAAGC
CCDC15.In8.3045F	CTATGCAGGCATCATCAGGC
CCDC15.In9.505F	CAATTCAGTTCCTCCTGGTC
CCDC15.In9.614R	CATCAGCCTCAGAAATCACTG
RELL2.In2.Py.Q.F	CACCTCCACTGACTCCCTC
RELL2.Ex3.Q.R	AGCTGCACTGTCCCTTCTC
Primers for editing examination	
CCDC15.In8.F	GAGAGAGGCACTTCCTCTGG
CCDC15.In9.R	GACTCCATCAGAAAAAAGCCAC
RELL2.In2.F	GCCCTGACCTCCACCTCCA
RELL2.In3.R	CCCTTCATGTTTTGTTTCTTACG
RNA probes	
CCDC15 In8 WT	CAUCAGAGGCAAGGUAGAAAAGAAAAAUGACAGAGAC CAA
CCDC15 In8 mut2	CAUCAGAGGCAAGGUAGAAGAGAAAAAUGACAGAGAC CAA
CCDC15 In8-9 WT	GGUAGAAAAGAAAAAUGACACUCGAGUGUUUUUUUCU CUUCUACUU
CCDC15 In8-9 Mut	GGUAGAAAAGAAAAAUGACACUCGAGUGUUAAAAACU CUUCUACUU
CCDC15 In8-9 WT (long)	CAUCAGAGGCAAGGUAGAAAAGAAAAAUGACAGAGAC CAAUUCCUCCUGGUCUCUCUCCUUUUUGUUUUUUCUC UUCUACUUUGUACUU
RELL2 Py-Ex3 WT	CCCUGACCUCCACCUCCACUGACUCCCUCUUUUCCUUCU UCCCUCAGCCAAUGCUGAGGCCUUGAAGGAGAUGCUGG GGGACAGUGAAGGAGAAGGGACAGUGCAGCUGUCCAG GUGAGCUG
RELL2 Py-Ex3 Mut	CCCUGACCUCCACCUCCACUGACUCCCUCUUUUCCUUCU UCCCUCAGCCAAUGCUGAGGCCUUGAACCACAUGCUGG

	GGGACAGUGAACCACAAGGGACAGUGCAGCUGUCCAGG UGAGCUG
