# Supplementary material for: Cis- and trans-regulations of pre-mRNA splicing by RNA editing enzymes influence cancer development
Source: Nat Commun. 2020 Feb 7;11:799. doi: 10.1038/s41467-020-14621-5 (PMC7005744; doi:10.1038/s41467-020-14621-5)
Supplement: Supplementary file 4 — Description of Additional Supplementary Files [file 41467_2020_14621_MOESM4_ESM.docx]

**Description of Additional Supplementary Files**

File name: Supplementary Data 1
Description: Input sequences for MEME
